# Supplementary material for: Integrative analysis of clinical and epigenetic biomarkers of mortality
Source: Aging Cell. 2022 May 12;21(6):e13608. doi: 10.1111/acel.13608 (PMC9197414; doi:10.1111/acel.13608)
Supplement: Supplementary file 1 — Fig S1‐3 [file ACEL-21-e13608-s001.docx]

**Supplementary Figure 1: Forest plots of the top CpGs in association with all-cause mortality in EA and AA cohorts.** A1-A10: top 10 CpGs identified in EA cohorts. B1-B10: top 10 CpGs identified in AA cohorts.

A1 A2

**
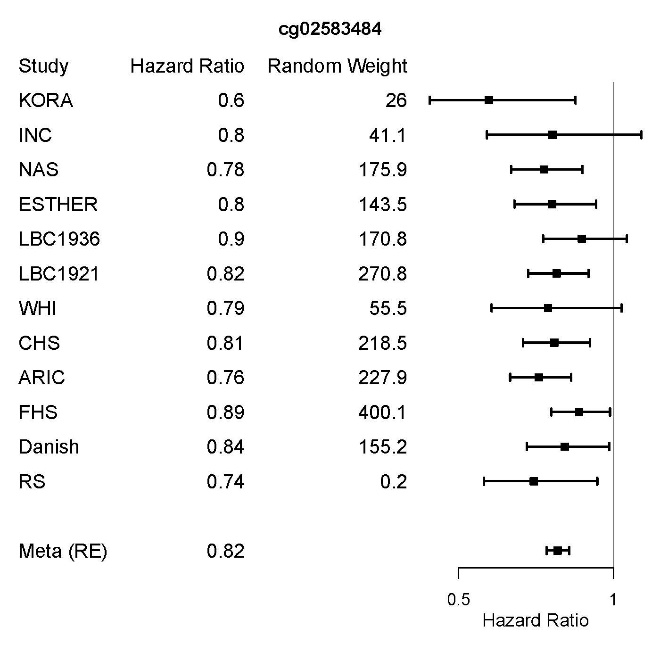

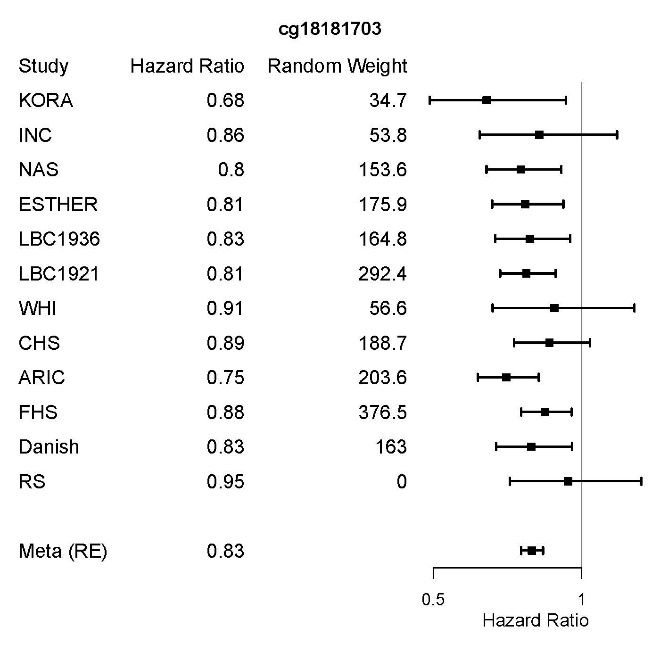
**

A3 A4

**
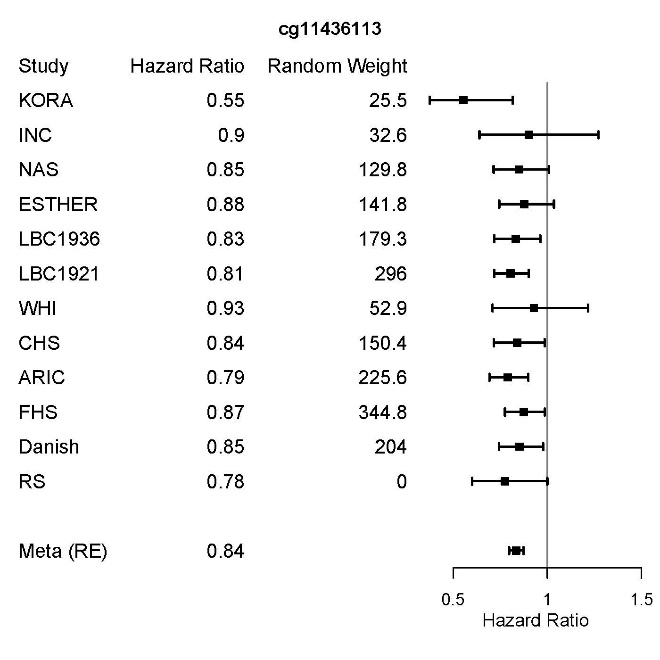
** **
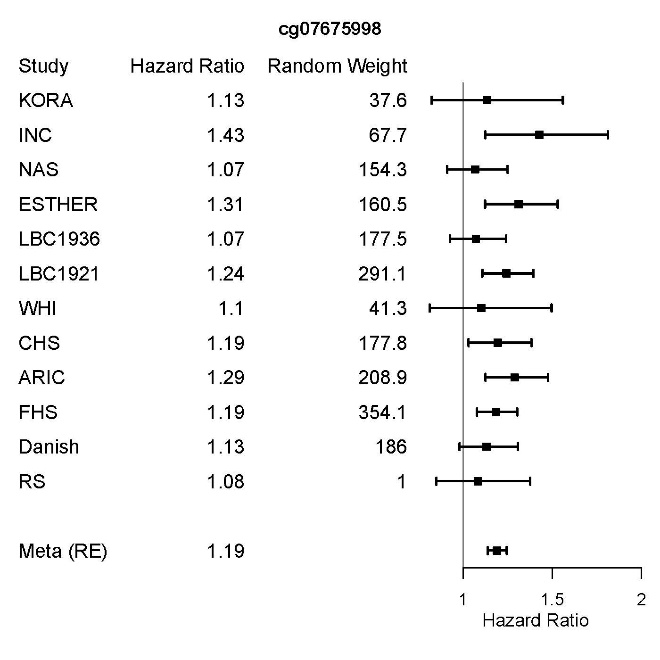
**

A5 A6

**
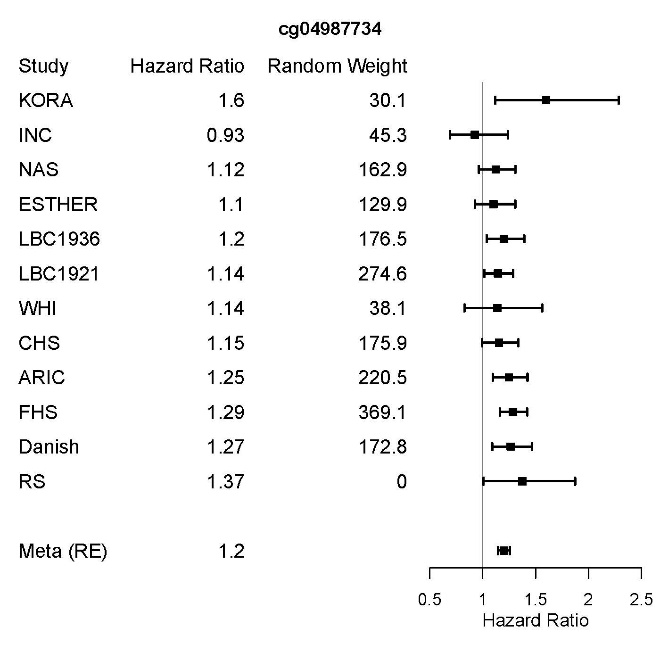
** **
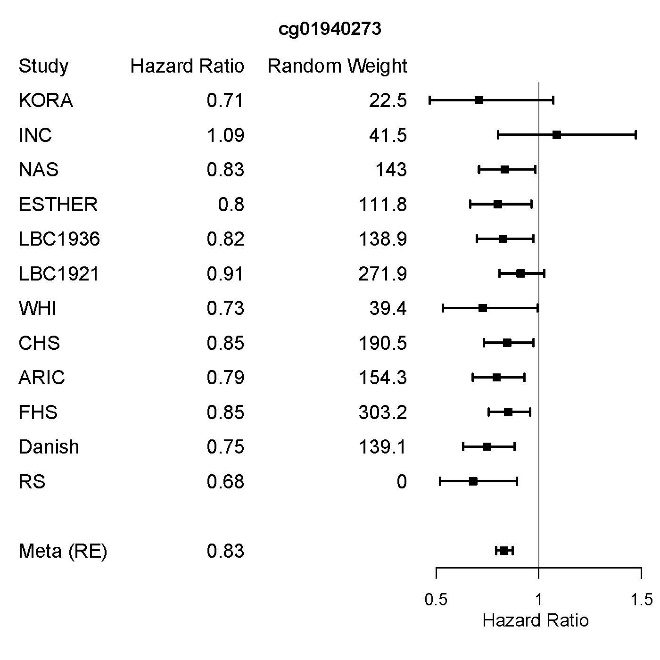
**

A7 A8

**
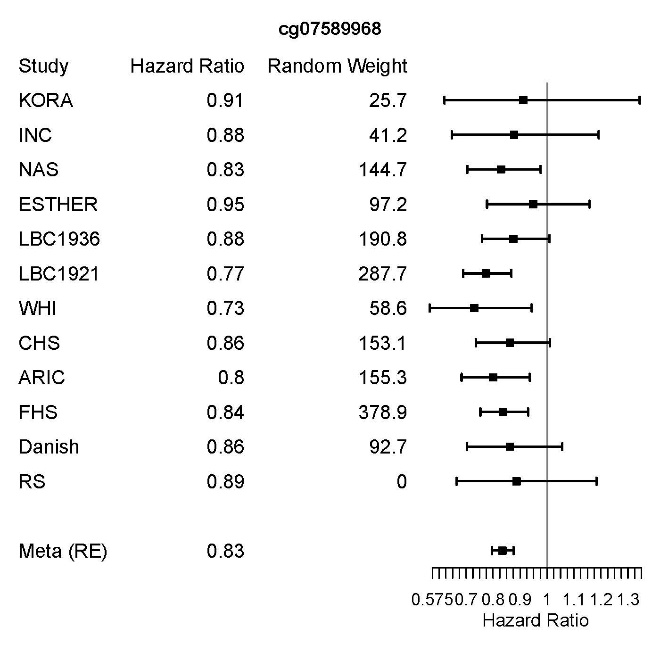
** **
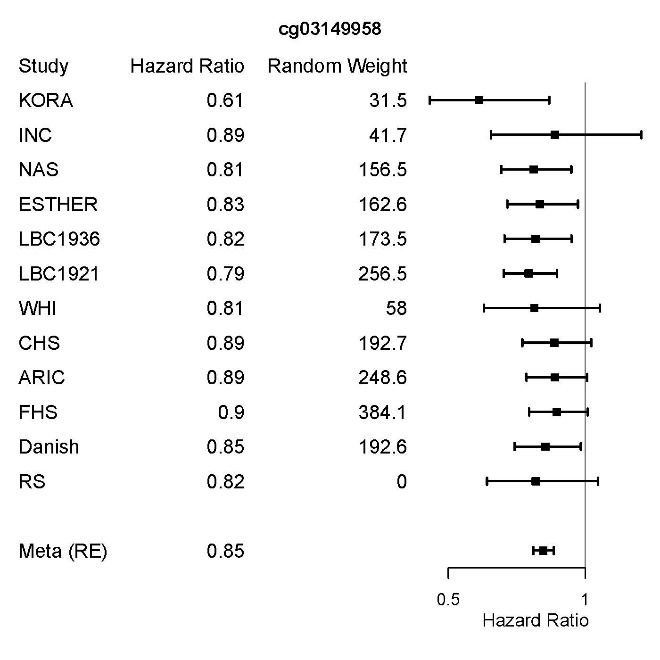
**

A9 A10

**
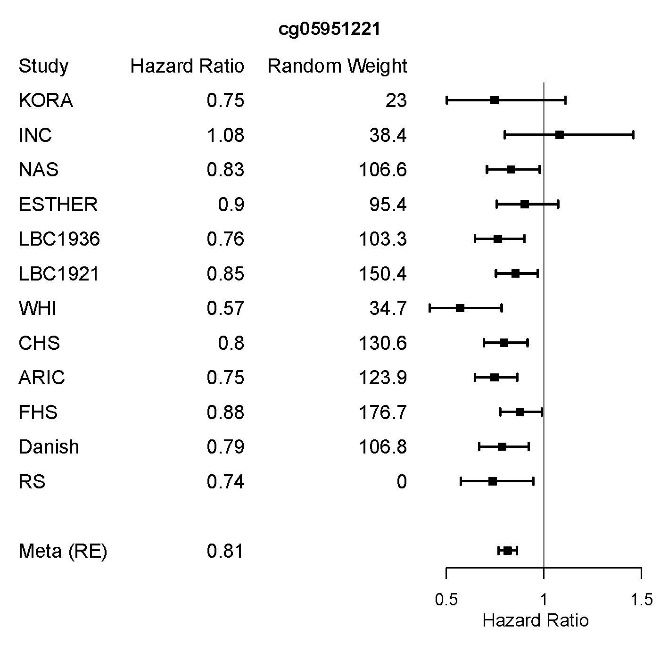

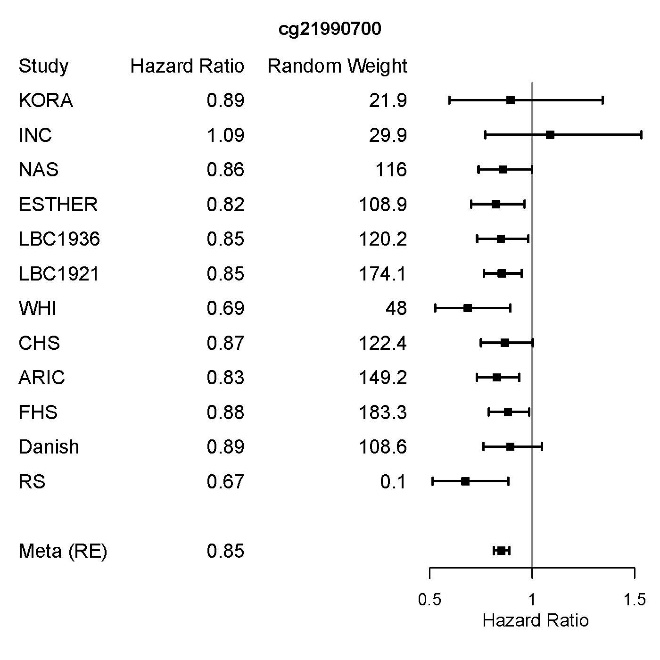
**

B1 B2


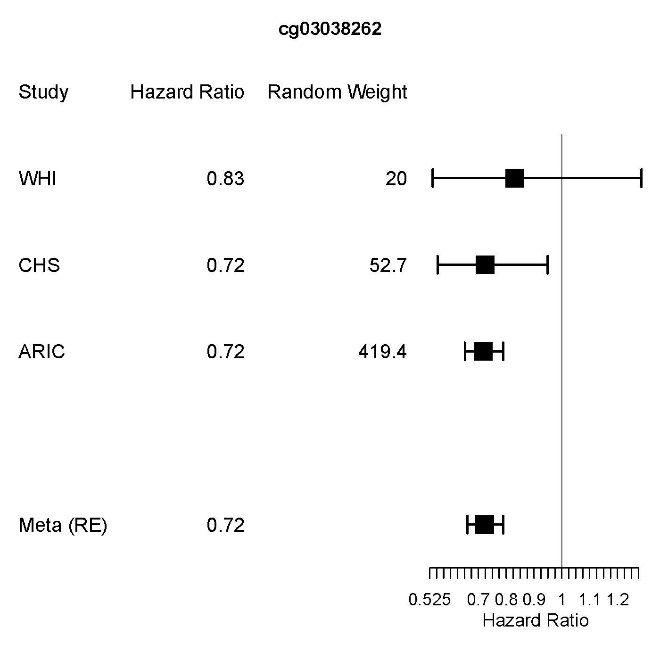

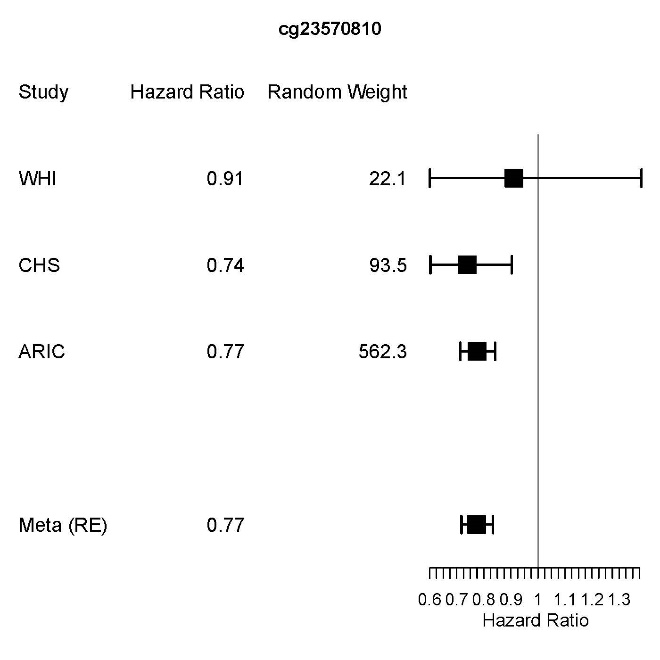


B3 B4


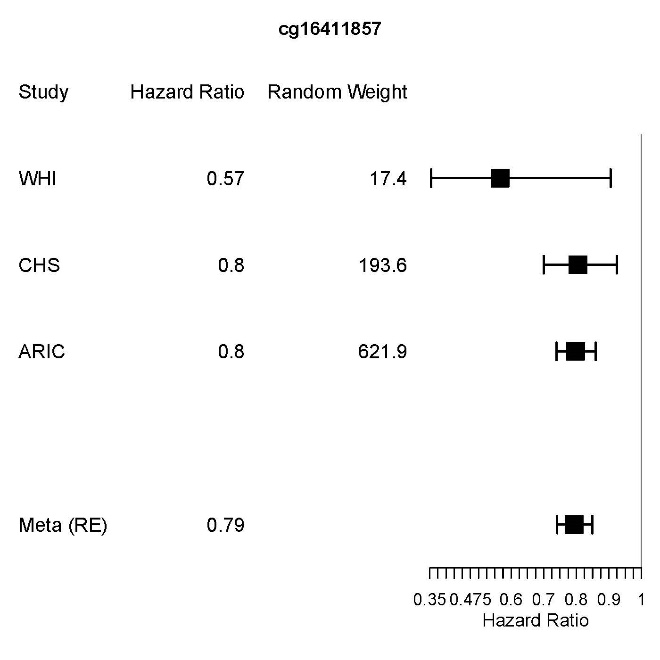

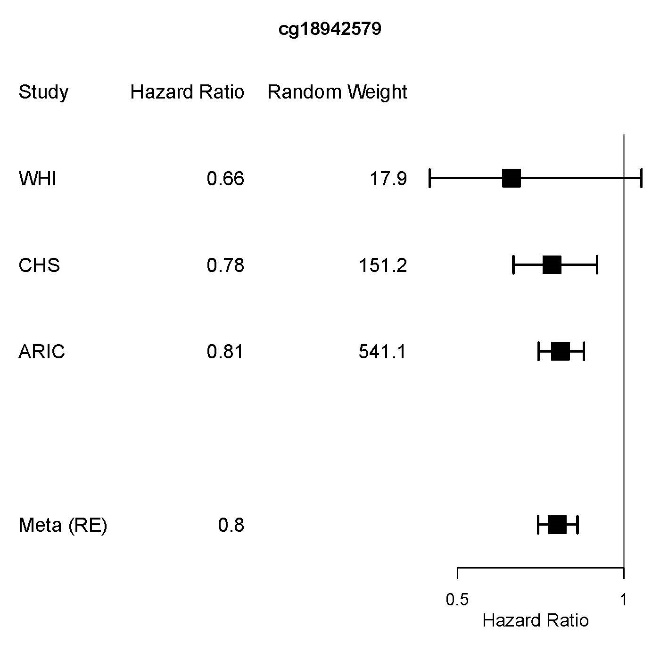


B5 B6


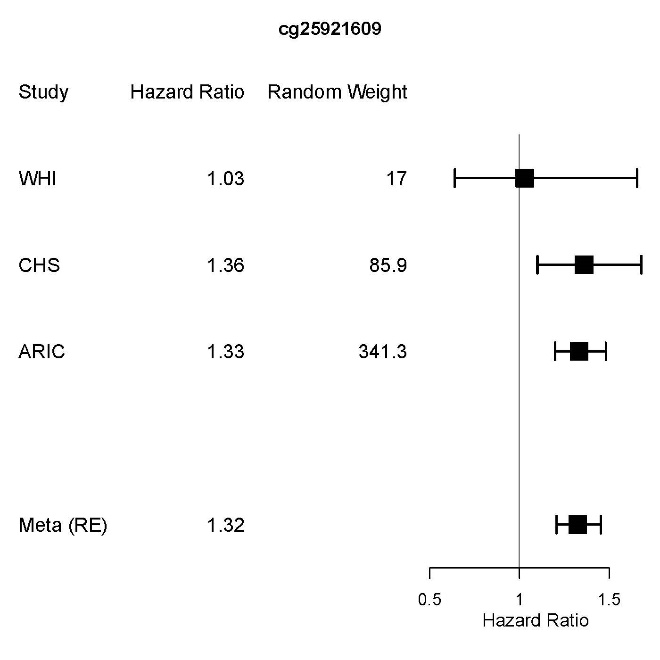

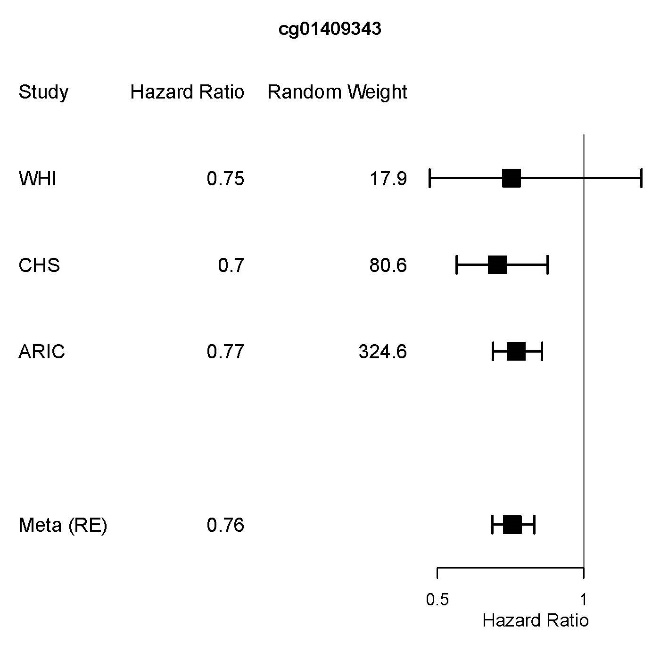


B7 B8


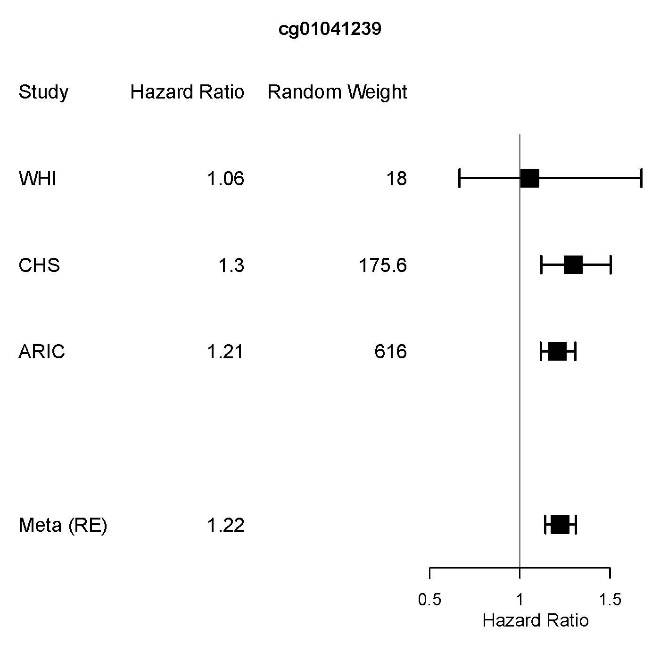

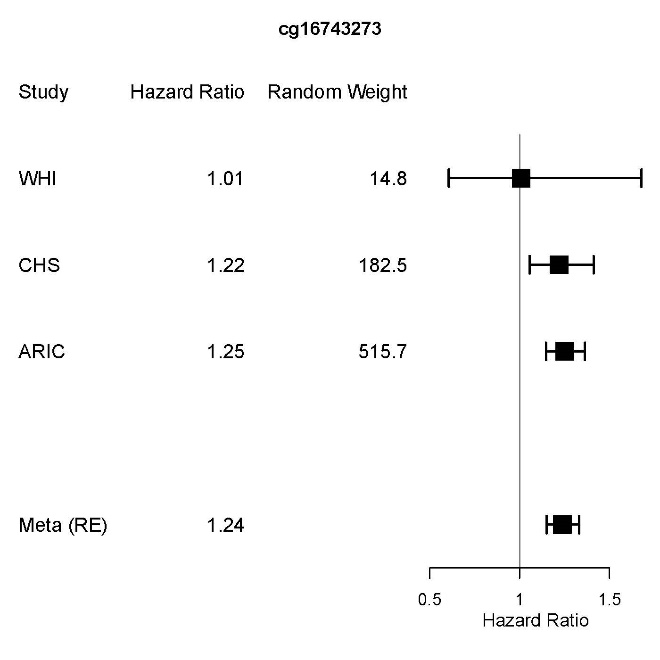


B9 B10


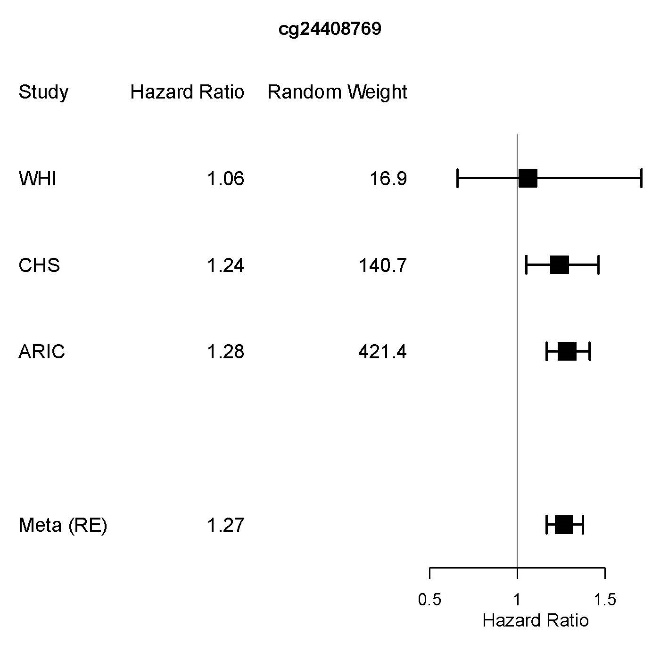

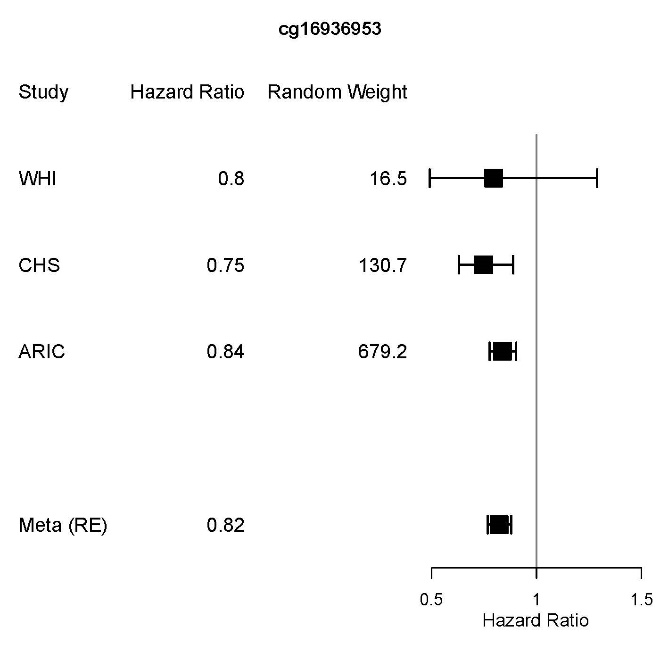


**Supplementary Figure 2: Comparison of beta values of CpGs for all-cause mortality with and without truncated at 15 years follow-up.**

**A B**

**Supplementary Figure 3: C-index of prediction models using different sets of input features.**  The prediction outcomes are A) all-cause mortality; B) CVD Death; and C) cancer death. The C-index values reflect the average values of 10 times cross-validation. For cancer death, the samples who were diagnosed any type of cancer before the time of whole blood draw for DNA methylation measurements were excluded when training and testing the model.

**A**

B

**C**
